# The clinical and psychological profiles of patients with hypogonadism, followed in 3 reference hospitals of Cameroon: an observational study

**DOI:** 10.11604/pamj.2019.33.47.18352

**Published:** 2019-05-22

**Authors:** Martine Claude Etoa Etoga, Gilbert Akwa, Anne Ongmeb Boli, Ahmadou Musa Jingi, Jean-Claude Njabou Katte, Nelly Sandra Ndam Ngambou, Brigitte Wandji, Mesmin Dehayem, Jean Claude Mbanya, Simeon-Pierre Choukem, Eugene Sobngwi

**Affiliations:** 1 Department of Clinical Sciences, Faculty of Medicine and Pharmaceutical Sciences, University of Douala, Douala, Cameroon; 2 National Obesity Center, Endocrinology and Metabolic Disease Unit, Yaoundé Central Hospital, Yaoundé, Cameroon; 3 Department of Internal Medicine and Specialties, Faculty of Medicine and Biomedical Sciences, University of Yaoundé 1, Yaoundé, Cameroon; 4 Internal Medicine Unit, Biyem-Assi District Hospital, Yaoundé, Yaoundé, Cameroon; 5 Department of Public Health, Faculty of Medicine and Biomedical Sciences, University of Yaoundé 1, Yaoundé, Cameroon; 6 Diabetes and Endocrine Unit, Yaoundé General Hospital, Yaoundé, Cameroon; 7 Gynecology and Obstetrics Unit, Yaoundé Central Hospital, Yaoundé, Cameroon; 8 Department of Internal Medicine, Faculty of Medicine and Pharmaceutical Sciences, University of Dschang, Dschang, Cameroon

**Keywords:** Hypogonadism, compulsive obsessive traits, Cameroon

## Abstract

**Introduction:**

Hypogonadism refers to a syndrome that results from failure of gonads to function properly. The main concern is considerable rise in morbidity, as shown by increased cardiovascular risk, infertility, osteoporosis and above all, the psychological impact on the life of the patients with hypogonadism. Judicious steroid replacement and culturally-sensitive psychological support before and during steroid therapy remains the key tool in the management of this condition. The present study aimed at filling the knowledge gap on hypogonadism in Cameroon.

**Methods:**

We conducted a cross-sectional study over a period of 12 months, in 3 reference hospitals of Cameroon. We included males and females diagnosed with hypogonadism, aged 16 to 50 years and 16 to 45 years respectively. After a complete clinical examination, patients were invited to fill the modified middlesex hospital questionnaire for psychoneurotic evaluation.

**Results:**

We recruited 59 patients with a sex ratio of 1:1. The mean age of the females and males were 27.7 ± 9.1years and 30.8 ± 11.7 years respectively. Normosmic Idiopathic Hypogonadotropic Hypogonadism (NIHH) was the most common presentation. Compulsive obsessive traits, phobic anxiety and hysterical trait, were most pronounced in these patients. Testosterone titers significantly correlated positively with testicular size and negatively with body mass index (BMI). A significant positive correlation was found between the testicular volumes measured with ultrasound (US) and with the orchidometer.

**Conclusion:**

Normosmic idiopathic hypogonadotropic hypogonadism is the most common presentation of hypogonadism in the study population. There is a significant psychosocial impact requiring further investigation and attention.

## Introduction

Hypogonadism refers to a syndrome that results from failure of gonadal function. It is a relatively rare disease that is underdiagnosed, with an unknown combined prevalence in both males and females worldwide [1]. Moreover, data is scarce in Africa. The most common presentation is delayed or absent puberty. Hypogonadism does not directly influence mortality but rather morbidity with an increased cardiovascular risk, infertility, osteoporosis and above all the psychological impact on the life of patients [2]. Psychological problems may go unnoticed if the physician does not address the subject first. In several African cultures, lack of adequate sex development is seen as a taboo. This may have a considerable sociocultural impact and cause stigmatisation among the patients. The principles of management of hypogonadism consist of sex steroid replacement and psychological support. Positive outcomes are obtained in most cases despite setbacks such as the risk of physical aggressiveness, increase sexual drive and suicidal tendencies [3-5]. Consequently, judicious steroid replacement and culturally-sensitive psychological support before and during steroid therapy remains the key tool in the management of this condition, especially in the context of limited access to health services and diagnostic methods of hypogonadism [6]. The present study aimed at filling the knowledge gap of hypogonadism in Cameroon.

## Methods

We conducted a cross-sectional study, over a period of 12 months, from January to December 2016. Our study was conducted in the Yaoundé Central and General hospitals; and at the Douala General hospital where patients with hypogonadism seen during outpatient visits were included. We invited people with hypogonadism-related symptoms through an announcement to consult an endocrinologist in one of the 3 study sites. For patients who already had follow-up in each unit, we recorded data from their files. All consenting patients received at outpatient endocrinology consultation with a diagnosis of hypogonadism and aged 16-50 years for males and or 16-45 years for females [7, 8] were included. Females who had undergone total hysterectomy and those with Mayer-Rokitansky-Küster-Hauser syndrome were excluded. We did a consecutive sampling.

### Data collection

**Clinical data:** for all patients we recorded medical history including cryptorchidism, mumps orchitis, impuberism, amenorrhea and their physiological age at the first consultation for hypogonadism-related symptoms. We also looked for symptoms such as anosmia and visual field defects. Family history of infertility or impuberism was also noted. Patients underwent a clinical exam where anthropometric parameters, eunuchoid presentation and specific signs of malformation such as web neck, narrow shoulders and cleft palates were assessed. Concerning external genital evaluation in men, we verified the presence of testes in the scrotum, measured testicular size with an orchidometer and the resting penile length using a tape then both were staged using the Tanner staging system. We evaluated and staged the female breast using the Tanner staging system. In both genders, pubic and axillary hair was evaluated for type and distribution and staged using the same staging system. Complete physical examination was conducted topographically.

**Psychosocial aspect:** we invited patients to fill a form that evaluated the direct effect of hypogonadism on their psychological state and the resultant psychosomatic disorder that could be associated with hypogonadism. We used the Middlesex Hospital Questionnaire (MHQ) that has been approved and considered valid, reliable [9, 10] and one of the most promising to be recommended in evaluating the psychological state of patients in non-psychiatric settings, appropriate for clinical and research practice [10]. This questionnaire has 48 questions and evaluates 6 psychological aspects: free floating anxiety, phobic anxiety, obsessive compulsive disorders, somatization, depression and hysterical traits. Each aspect is evaluated by 8 questions rated on a scale of 0 to 2.

**Data analysis:** data were collected and analyzed using SPSS software version 23. Student T-test was used to compare means and Spearman's correlation to establish relationship between qualitative variables. The results were expressed in terms of percentages for qualitative variables, medians and means for quantitative variables. The threshold of significance was set at a value p < 0.05.

**Ethical consideration:** institutional ethical clearance was obtained from the Ethical committee of the Faculty of Medicine and Biomedical Sciences of the University of Yaoundé I to carry out this study. Each participant gave his informed written consent. They were free to withdraw from the study at any time they wished. All information obtained or used in the study was kept confidential.

## Results

We recruited 59 patients including 30 females aged 29.0 ± 9.1and 29 males aged 30.8 ± 11.7 years. The majority (69.5%) of our patients were single. The majority of our population had body mass index (BMI) above normal. In females, 33.3% (10) had BMI between 25 and 30 kg/m^2^ and 33.3% (10) also had BMI between 30 and 35 kg/m^2^. Whereas in the male population 34.5% (10) had BMI between 25 and 30 kg/m^2^ and 34.5% (10) also had BMI between 30 and 35 kg/m^2^. The main presenting complaints in the females were primary amenorrhoea 53.3% (16) and secondary amenorrhoea 33.3% (10) (Table 1). In addition, 83.3% (15) had decreased libido, 63.3% (19) had primary amenorrhoea, 53.3% had sleep disorders, 53.3% had emotional lability, 50.0% (15) had irritability, 46.7% (14) had vaginal dryness, 40.0% (12) had secondary amenorrhoea and 72.2% (13) of those who desired conception were infertile. As regards clinical signs, 76.7% (23) had eunuchoid habitus, most were either in Tanner stage 3 or 4 for pubic hair development accounting for 36.7% (11) and 30.0% (9) respectively, whereas the majority of women were in either Tanner stage 5 or 3 for breast development accounting for 36.7% (11) and 30.0% (9) respectively. The only physical syndrome identified in females was Turner syndrome.

**Table 1:** Distribution of presenting complaint

Gender	Proposition	Frequency	Percentage (%)
**Female**	Primary amenorrhoea	16	53.3
	Secondary amenorrhoea	10	33.3
	Short statue	5	16.7
	Anosmia	0	0.0
	Inability to conceive	3	10.11
	Small breasts	5	16.7
	Others	2	6.6
**Male**	Absence beard on chin	8	27.6
	Micro-penis	14	48.3
	Cryptorchidism	3	10.3
	Small testes	11	37.9
	Short stature	4	13.8
	Gynecomastia	3	10.3
	Erectile dysfunction	2	6.9
	Anosmia	0	0.0
	Inability to impregnate a woman	3	10.3
	Others	8	27.6

In the male population, micro-penis accounted for 48.3% (14) and small testes for 37.9% (11) of the presenting complaints. In addition, 75.9% (22) had sleep disorder, 72.4% (21) had both erectile dysfunction and low libido, 55.2% (16) had fatigue, 44.8% (13) irritability, 41.4% (12) had a high pitched voice and 14 had infertility. Concerning clinical signs, 79.3% (23) had eunuchoid habitus, 69.0% (20) lacked beard and most were in Tanner stage 3 or 4 for pubic hair development accounting for 34.5% (10) and 31.0% (9) respectively. On the other hand, 72.4% of the males had a penile length in Tanner stage 5, 62.1% (18) had gynecomastia. Kallman and Klinefelter syndromes were identified. In the overall population, the most common diagnosis was congenital hypogonadotropic hypogonadism seen in 44.1% (26) of the patients. Congenital hypergonadotropic hypogonadism was found in 22.0% (13), 18.6% (11) had acquired hypogonadotropic hypogonadism meanwhile 15.3% (9) had acquired hypergonadotropic hypogonadism. In female patients, the main etiology of central hypogonadism was NIHH with a prevalence of 20.0% (6). Peripheral or primary etiologies included primary ovarian failure (POF) accounting for 20.0% (6), Turner syndrome accounting for 16.7% (5), prolactinomas accounting for 13.3% (4) and pure gonadal dysgenesis in 10.0% (3) of the cases. On the other hand, male patients presented with NIHH in 34.5% (10) of the cases and 23.8% (4) of cases were part of the combined pituitary hormone deficiency syndrome (Table 2).

**Table 2:** Causes of hypogonadism

Gender	Causes	Frequency
**Female**		
	Normosmic Idiopathic Hypogonadotropic Hypogonadism	20
	Part of Combined Pitutary Hormon Deficiency	3.3
	Turner syndrome	16.7
	Pure gonadal dysgenesis	10
	Ovarian agenesis	3.3
	Sewyer syndrome	6.7
	Prolactinoma	13.3
	Pituitary apoplexia	3.3
	Premature Ovarian Failure	20
	Bilateral ovarectomy	3.3
**Male**		
	Kallman Syndrome	13.8
	Normosmic Idioapthic Hypogonadotropic Hypogonadism	34.5
	Part of the Combined Pitutary Hormonal Deficiency	23.8
	Klinefelter syndrome	6.9
	Craniopharyngioma	3.4
	Prolactinoma	6.9
	Non-secreting macroadenoma	3.4
	Chronic orchitis	6.9

**Correlation studies:** there was a significant positive correlation between testicular volume measured using the orchidometer and that measured by testicular ultrasound with a correlation coefficient 0.860 and p-value < 0.001 on both sides. There was a positive correlation between the BMI and the level of androgens in both sexes.

**Psychological profile:** concerning the psychological profile in males, compulsive obsessive trait was common with a mean score of 8.6 ± 3.4 followed by phobic anxiety and depression with scores 7.8 ± 2.9. Meanwhile women presented a score of 9.2 ± 2.5 for compulsive obsessive trait, followed by phobic anxiety with a mean score 8.7 ± 2.7 and the minimum mean was 6.9 ± 2.1 for hysterical trait (Table 3).

**Table 3:** Psychological characteristics

Gender	Trait	Mean±SD (Reference)	Mean± SD (our patients)	P-value
**Male**	Free-floating anxiety	5.1±3.1	7.7±3.1	0.04
	Phobic anxiety	2.9±2.2	7.8±2.9	0.003
	Obsessive compulsive trait	5.8±3.1	8.6±3.4	0.12
	Somatization	3.2±2.4	7.1±2.7	0.0005
	Depression	3.3±2.5	7.8±2.5	0.02
	Hysteria	7.5±3.1	6.2±2.3	0.03
**Female**	Free-floating anxiety	5.1±3.1	7.6±3.2	0.04
	Phobic anxiety	2.9±2.2	8.7±2.7	0.003
	Obsessive compulsive trait	5.8±3.1	9.2±2.5	0.12
	Somatization	3.2±2.4	7.9±3.3	0.0005
	Depression	3.3±2.5	8.0±2.3	0.02
	Hysteria	7.5±3.1	6.9±2.1	0.03

## Discussion

This cross-sectional and observational study on hypogonadism in reference centers of a low resource setting shows the burden of late diagnosis, as the main presenting complaint was delayed or absent puberty and marked deteriorated psychological profiles in both sexes. Most patients consulted late given that the main complaint was delayed puberty and their mean age was 30.8 ± 11.5 years, for men and 27.7 ± 9.1 years for women. Delayed puberty is defined as the absence of secondary sexual characteristics or any signs of puberty beyond the normal age range for a given population. The common setpoint is 13 years in girls and 14 in boys [11]. Carel *et al.* found an age of 22.7 ± 2.6 years in patients with Turner syndrome [12]. This age is lower than ours. However, another study conducted in our setting by Wonkam *et al.* found a mean age 18.4 ± 2.8 years at diagnosis of Turner syndrome in a population of patients followed in Yaoundé [13]. Although both studies were conducted in Cameroon, the age difference could be due to the fact that Wonkam *et al.* worked on a pediatric population. Patients reported late because the condition was not perceived as being pathologic. Hence these developmental abnormalities could go unnoticed or be neglected by parents or the patients themselves. Generally, it is at adulthood that the patients seeked the attention of a health professional because of the absence of secondary sexual characteristics, amenorrhoea in the context of a new relationship or the desire to procreate. Moreover, poor road infrastructure, low socio-economic backgrounds, illiteracy ignorance could contribute to the delay of diagnosis [14].

In contrast to literature that suggests hypergonadotropic hypogonadism as being more common than hypogonadotropic hypogonadism [1], NIHH was the main cause of hypogonadism in our population. This could be explained by the fact that our population was relatively small, and hospital based rather than community based. Some specificities were observed such as one patient presenting with a history of cleft palate, cleft lip and a normal sense of smell, suggesting a mutation of fibroblast growth factor receptor 1 (FGFR1) [15] or a mutation in FGF8 [16]. Furthermore, we observed in line with literature a stronger correlation between testicular volume measured by ultrasound and that measured by the orchidometer [17]. Thus, the orchidometer which is accessible, convenient and easy to use could be a promising tool for clinical practice and patient follow up in our resource-limited setting. Concerning the psychological profile of the patients, mean scores for phobic anxiety, obsessive compulsive disorders, depression and hysteria were greater than the standardized mean scores in psychoneurotic patients at psychiatrist consultation in contrast with some studies of Western countries. Georgopoulos *et al.* found a satisfying psychological profile in congenital hypogonadotropic hypogonadism patients compared to a control group [18]. This result reflects the low acceptability of the condition in our cultural context that may worsen the psychological profile and further explain delayed consultation. Testosterone is known to have organizational, neurotropic and neuroprotective effects on the brain thus lack of androgen could contribute to the poor psychological profile we observed especially in primary hypogonadism and in Klinefelter Syndrome cases in particular [19].

## Conclusion

Normosmic idiopathic hypogonadotropic hypogonadism (NIHH) is the most common presentation of hypogonadism in the study population. The clinical presentation of hypogonadism is heterogeneous and there is a significant psychosocial impact requiring further investigation and attention.

### What is known about this topic

- Hypogonadism is a frequent complain in endocrine consultation;
- The etiologies are not different in both genders;
- Spectrum of anosmia is widely variable in hypogonadism.

### What this study adds

- Hypogonadotropic hypogonadism is the most frequent forms of hypogonadism in our context;
- The study highlights a delay diagnostic in our context and the clinical presentation at the end stage of the disease, difficulty and the limits of the management of hypogonadism in a low resource setting;
- It presents specific psychiatric disorders in all types of hypogonadism and importance to systematically implicate psychiatrists and psychologists in the overall management of hypogonadic patients in our context.

## Competing interests

The authors declare no competing interests.
